# A multi-task learning model using RR intervals and respiratory effort to assess sleep disordered breathing

**DOI:** 10.1186/s12938-024-01240-0

**Published:** 2024-05-05

**Authors:** Jiali Xie, Pedro Fonseca, Johannes van Dijk, Sebastiaan Overeem, Xi Long

**Affiliations:** 1 Biomedical Diagnostics Lab, Department of Electrical Engineering, Eindhoven University of Technology, 5612 AZ Eindhoven, The Netherlands; 2 Philips Research, High Tech Campus, 5656 AE Eindhoven, The Netherlands; 3 Sleep Medicine Center Kempenhaeghe, 5591 VE Heeze, The Netherlands; 4 Department of Orthodontics, Ulm University, 89081 Ulm, Germany; 5 Department of Electrical Engineering, Eindhoven University of Technology, PO Box 513, 5600 MB Eindhoven, The Netherlands; 6 Eindhoven MedTech Innovaton Center (e/MTIC), P.O. Box 513, 5600 MB Eindhoven, The Netherlands

**Keywords:** Obstructive sleep apnea, Multi-task learning, Deep learning, Machine learning, Insomnia

## Abstract

**Background:**

Sleep-disordered breathing (SDB) affects a significant portion of the population. As such, there is a need for accessible and affordable assessment methods for diagnosis but also case-finding and long-term follow-up. Research has focused on exploiting cardiac and respiratory signals to extract proxy measures for sleep combined with SDB event detection. We introduce a novel multi-task model combining cardiac activity and respiratory effort to perform sleep–wake classification and SDB event detection in order to automatically estimate the apnea–hypopnea index (AHI) as severity indicator.

**Methods:**

The proposed multi-task model utilized both convolutional and recurrent neural networks and was formed by a shared part for common feature extraction, a task-specific part for sleep–wake classification, and a task-specific part for SDB event detection. The model was trained with RR intervals derived from electrocardiogram and respiratory effort signals. To assess performance, overnight polysomnography (PSG) recordings from 198 patients with varying degree of SDB were included, with manually annotated sleep stages and SDB events.

**Results:**

We achieved a Cohen’s kappa of 0.70 in the sleep–wake classification task, corresponding to a Spearman’s correlation coefficient (*R*) of 0.830 between the estimated total sleep time (TST) and the TST obtained from PSG-based sleep scoring. Combining the sleep–wake classification and SDB detection results of the multi-task model, we obtained an *R* of 0.891 between the estimated and the reference AHI. For severity classification of SBD groups based on AHI, a Cohen’s kappa of 0.58 was achieved. The multi-task model performed better than a single-task model proposed in a previous study for AHI estimation, in particular for patients with a lower sleep efficiency (*R* of 0.861 with the multi-task model and *R* of 0.746 with single-task model with subjects having sleep efficiency < 60%).

**Conclusion:**

Assisted with automatic sleep–wake classification, our multi-task model demonstrated proficiency in estimating AHI and assessing SDB severity based on AHI in a fully automatic manner using RR intervals and respiratory effort. This shows the potential for improving SDB screening with unobtrusive sensors also for subjects with low sleep efficiency without adding additional sensors for sleep–wake detection.

## Background

Sleep-disordered breathing (SDB) stands out as one of the most prevalent sleep disorders. It is characterized by partial (hypopnea) or complete (apnea) obstruction of the upper airway during sleep. The severity of SDB is commonly quantified through the apnea–hypopnea index (AHI), which represents the number of apnea and hypopnea events per hour of sleep [1]. This disorder has been associated with various adverse consequences, including daytime sleepiness, memory impairment, hypertension, cardiovascular ailments, and stroke [2–5]. It is estimated that, among adults aged between 30 and 70 years, approximately 13% of men and 6% of women exhibit moderate to severe SDB (AHI ≥ 15) and the incidence of SDB is on the rise worldwide [6], underscoring the urgency for research in this field.

Currently, the ultimate gold standard for diagnosing SDB remains a full night polysomnography (PSG), and combining the measurement of neurophysiological signals, to measure sleep, and respiratory signals such as airflow, peripheral oxygen saturation (SpO_2_) and respiratory effort, to assess SDB [7]. However, PSG has several limitations, such as high cost, substantial labor requirements, patient discomfort due to attached sensors, and long waiting lists, all of which hinder its accessibility. In response to these challenges, polygraphic home sleep apnea tests, which comprise a smaller set of sensors typically restricted to the measurement of respiratory signals, have emerged as an alternative for SDB diagnosis [8, 9]. Nevertheless, even these home tests still demand significant labor input and are financially burdensome, often requiring qualified technicians to set up the equipment [10]. Moreover, both PSG and home tests often require manual scoring, or at least manual confirmation after automatic scoring, and are practically limited to one or two nights of measurement. Importantly, they are typically only available on referral after suspicion of clinically relevant SDB. Longer-term multi-night testing, and follow-up testing are not practically feasible with existing diagnostic tools. Hence, there is a pressing need for a readily available, cost-effective, and automated approach to monitor SDB for broader public and clinical use.

Various approaches have been studied as alternatives to standard signals used to score SDB and estimate AHI. For instance, Saha et al. [11] employed thresholds on features derived from arterial oxygen saturation measured by pulse oximeter, respiratory sounds, and movement to detect SDB events. Their method showed an F1-score ranging from 0.22 ± 0.10 for AHI ≤ 5 to 0.72 ± 0.09 for AHI > 30, with a coefficient of determination *R*^2^ of 0.88 between the estimated AHI and the reference AHI from PSG. Deviaene et al. [12] utilized a random forest classifier on time-domain features derived from oxygen saturation (SpO_2_) signals to classify apnea and hypopnea events during all SpO_2_ desaturation events, achieving a detection accuracy of 82.8%. They reported *R*^2^ values ranging from 0.86 to 0.95 for AHI estimation, using different datasets. In another study, Kokkalas et al. [13] applied convolutional recurrent neural networks on electroencephalographic signals to detect apnea and hypopnea events, obtaining a sensitivity of 0.73, a positive predictive value of 0.78, and a correlation coefficient *R* of 0.88 for AHI estimation. Olsen et al. [14] proposed a method based on the detection of RR intervals from electrocardiography (ECG), and ECG-derived respiration (EDR) using recurrent neural network (RNN) for detecting SDB events. Their approach achieved an overall sensitivity of 70.9%, a precision of 73.4%, and an F1 score of 72.1% for event detection, along with an *R*^2^ of 0.828 for AHI estimation. In our previous research [15], we replaced the EDR with respiratory effort (RE) measured by respiratory inductive plethsmography (RIP) belts in the model from Olsen et al. [14] and evaluated this modified approach with a new dataset, finding that RE outperformed EDR when combined with ECG for SDB event detection. Specifically, we achieved improved performance in both SDB event detection (F1 score was improved from 0.607 to 0.708) and AHI estimation (Spearman’s correlation coefficient R was improved from 0.904 to 0.922).

A recurrent issue observed in these studies pertains to the AHI estimation, which is based either on total recording time (TRT) [11–13] or total sleep time (TST) [14, 15] derived from manual annotation obtained from PSG. Using TRT to estimate AHI may introduce underestimation problems, particularly due to a substantial proportion of individuals with SDB also experiencing comorbid insomnia [16–18], which reduces the fraction of TST with respect to the TRT. Employing manual sleep annotations derived from PSG for AHI estimation gives rise to practical deployment issues.

In our previous study, we used RR intervals from ECG and RE from RIP belts, together with manual scoring of sleep and wake periods, and achieved high performance in AHI estimation [15]. In the present study, we address the aforementioned limitation by introducing a multi-task model using the same inputs. This model concurrently detects SDB events and performs sleep–wake classification. The proposed approach can simultaneously provide an estimation of TST, the number of SDB events, and consequently, AHI, without necessitating additional signals, or manual sleep annotation.

## Results

### Sleep–wake classification

**Fig. 1:**
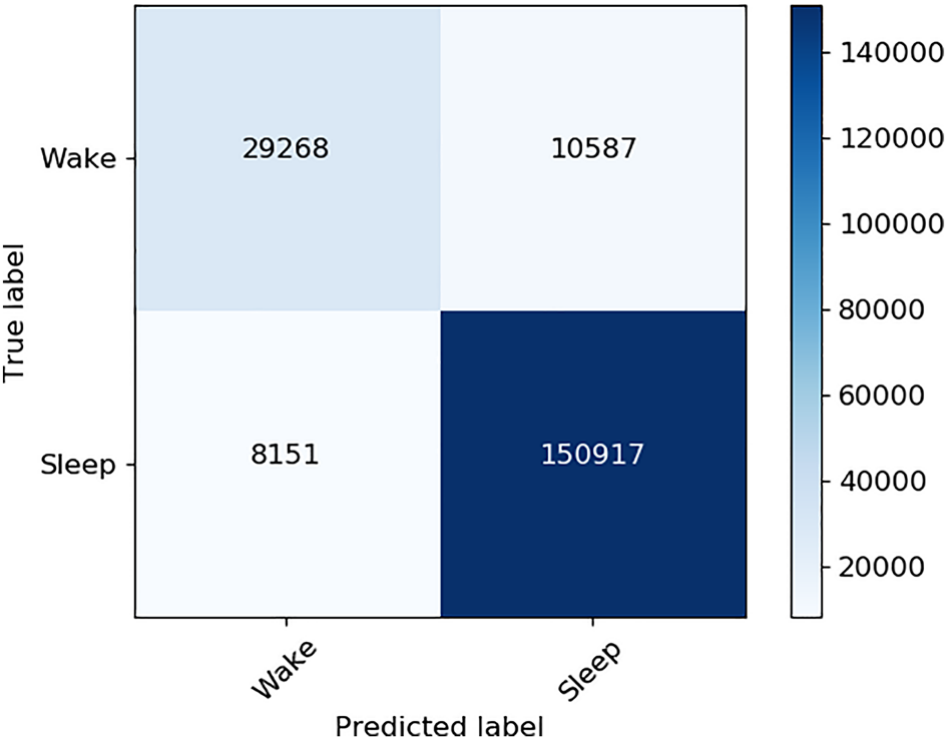
Confusion matrix for sleep–wake classification

**Fig. 2:**
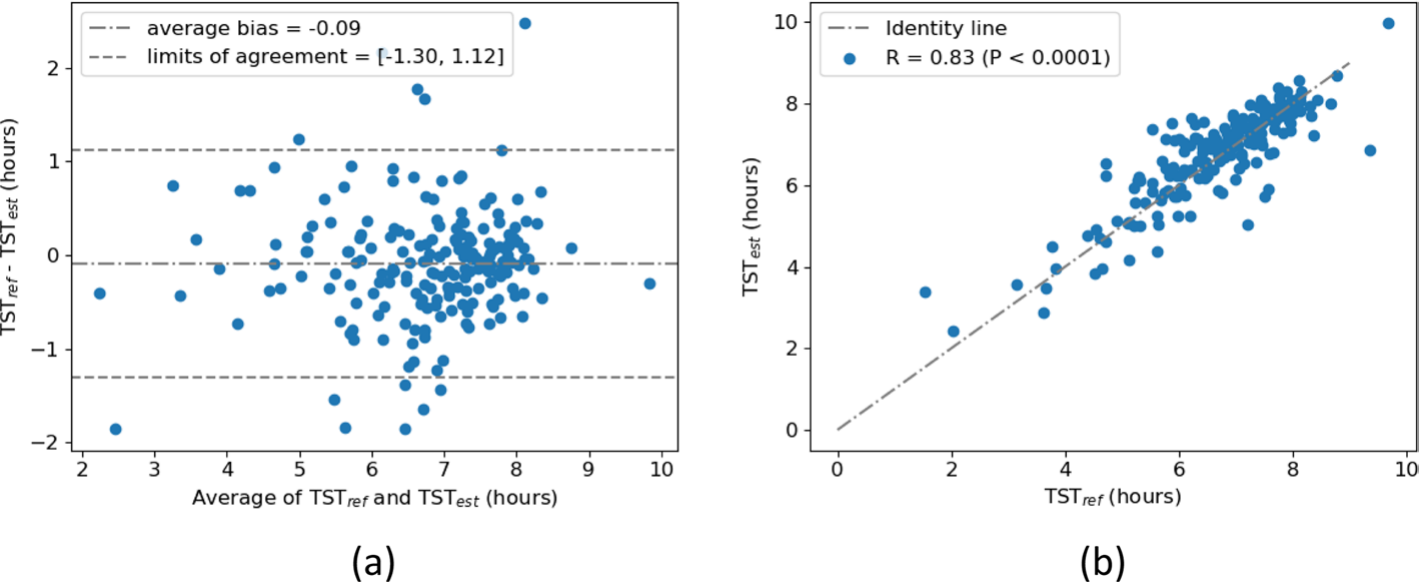
TST estimation results. **a** Bland–Altman plot between TST_ref_ and TST_est_. **b** Scatter plot between TST_ref_ and TST_est_

Sleep–wake classification achieved F1 scores of 0.942 (mean ± standard deviation across subjects: 0.936 ± 0.055) and 0.758 (0.719 ± 0.130) considering sleep as positive class, and wake as positive class, respectively. The sensitivity was 0.949 for sleep as positive class (0.946 ± 0.061) and 0.734 for wake (0.724 ± 0.164). The specificity was 0.934 for sleep as positive class (0.930 ± 0.072) and 0.782 for wake (0.770 ± 0.178). The Cohen’s kappa for the classification of all 30-s epochs into sleep and wake in comparison with scorings from PSG was 0.70 (0.66 ± 0.14 per subject). The confusion matrix obtained after aggregating all epochs from all testing recordings is presented in Fig. 1. Figure 2 illustrates Bland–Altman and the scatter plot for TST estimation. With a Spearman’s correlation coefficient of *R* = 0.83 (*P* < 0.0001) and a mean absolute error (MAE) of 0.445 between the reference TST (TST_ref_) and the estimated TST (TST_est_), the classifier slightly overestimated TST by 0.09 h, with 95% limits of agreement of [− 1.30, 1.21] hours.

### SDB event detection

Table 1 presents the SDB event detection results both in terms of the mean and standard deviation (SD) per subject, as well as the aggregated (pooled) outcomes from events from all recordings. Overall event detection performance achieved an F1 score of 0.631.

**Table 1:** SDB event detection results

	Sensitivity (%)	Precision (%)	F1 score
Mean ± SD	53.1 ± 23.9	50.6 ± 22.3	0.484 ± 0.216
Pooled	63.0%	63.3%	0.631

Sample statistics indicate per-subject mean ± standard deviation

### AHI estimation

**Fig. 3:**
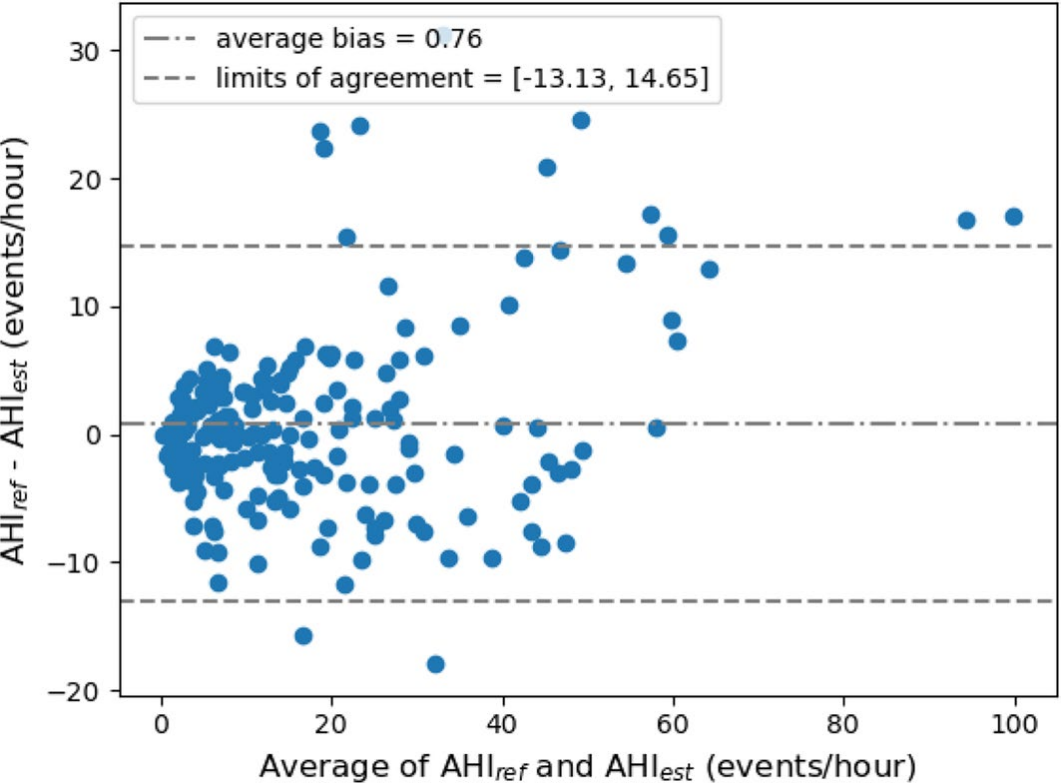
Bland–Altman plot between AHI_ref_ and AHI_est_

**Fig. 4:**
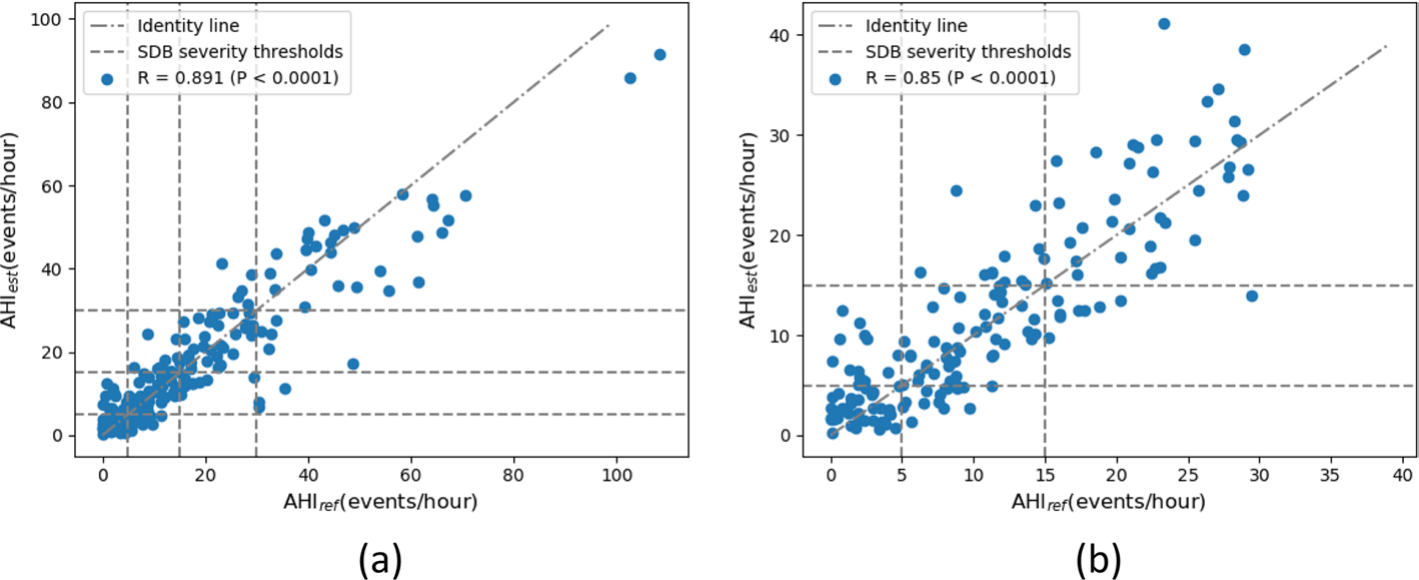
Scatter plots. **a** All AHI range. **b** AHI below the threshold of severe SDB (AHI_ref_ < 30)

The multi-task model achieved a Spearman’s correlation coefficient between AHI_ref_ and AHI_est_ of 0.891 (*P* < 0.0001), with a small underestimation bias of 0.76 events/h, and 95% limits of agreement of [− 13.13, 14.65] event/hour. Figure 3 depicts the Bland–Altman analysis and Fig. 4 shows the scatter plot with the AHI estimation results, for both the complete AHI range, as well as with the range of AHI below the threshold of severe SDB (AHI < 30). For this smaller range, the model achieved a Spearman’s correlation coefficient of 0.850 (*P* < 0.0001).

### SDB severity classification

**Fig. 5:**
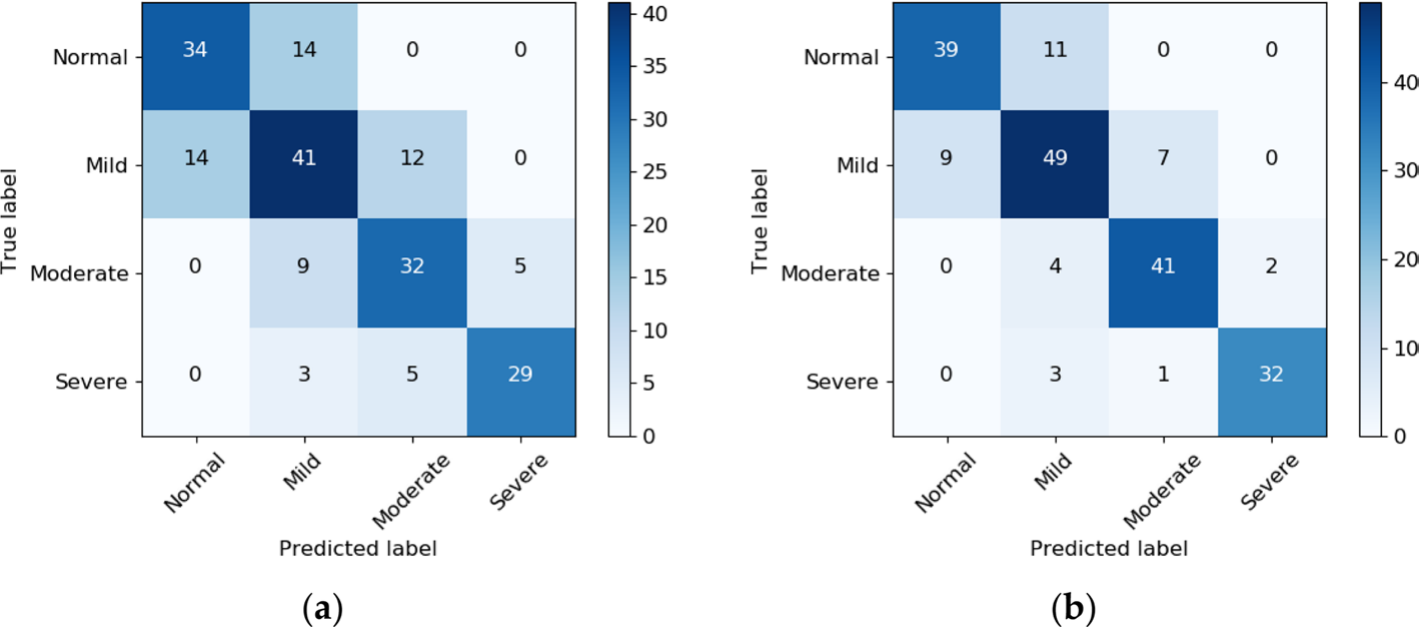
Confusion matrix of SDB severity classification based on AHI. **a** Without NBL. **b** With NBL

Using the classical thresholds for SDB severity classification the model achieved an accuracy of 68.7% and a kappa of 0.58 Fig. 5(a). Using near-boundary double-labeling (NBL), performance increased to an accuracy of 81.3% and a kappa of 0.75, reflecting that a large percentage of misclassifications occur near the classical severity thresholds.

### Comparison between model with and without sleep–wake classification

**Fig. 6:**
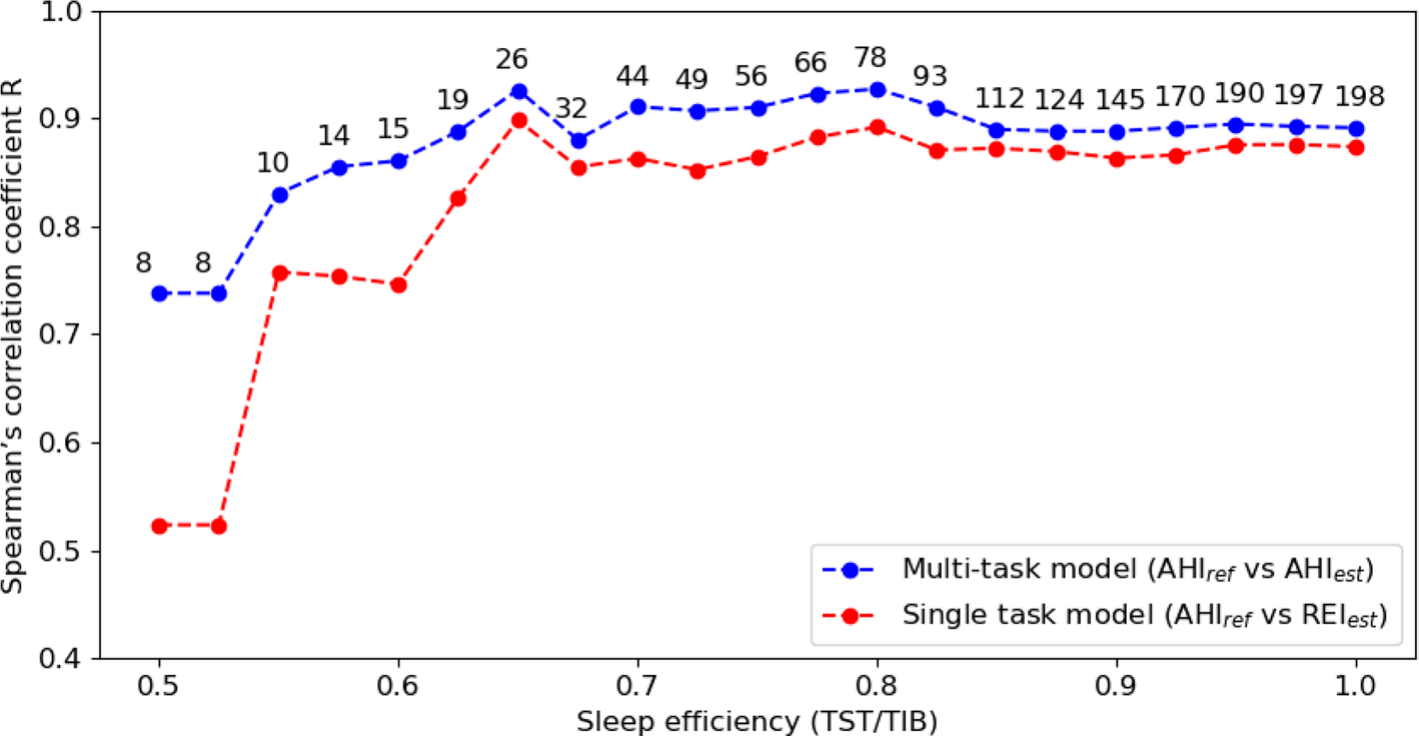
Spearman’s correlation coefficient between AHI_ref_ and AHI_est_ for the model with sleep–wake classification, and between AHI_ref_ and REI_est_ for the model without sleep–wake classification, both as a function of sleep efficiency. Each data point in the figure represents the coefficient calculated for subjects with sleep efficiency lower than or equal to the corresponding value. Additionally, the number above each point indicates the total number of subjects included in the respective analysis. *TST:* total sleep time, *TIB:* total time in bed

**Fig. 7:**
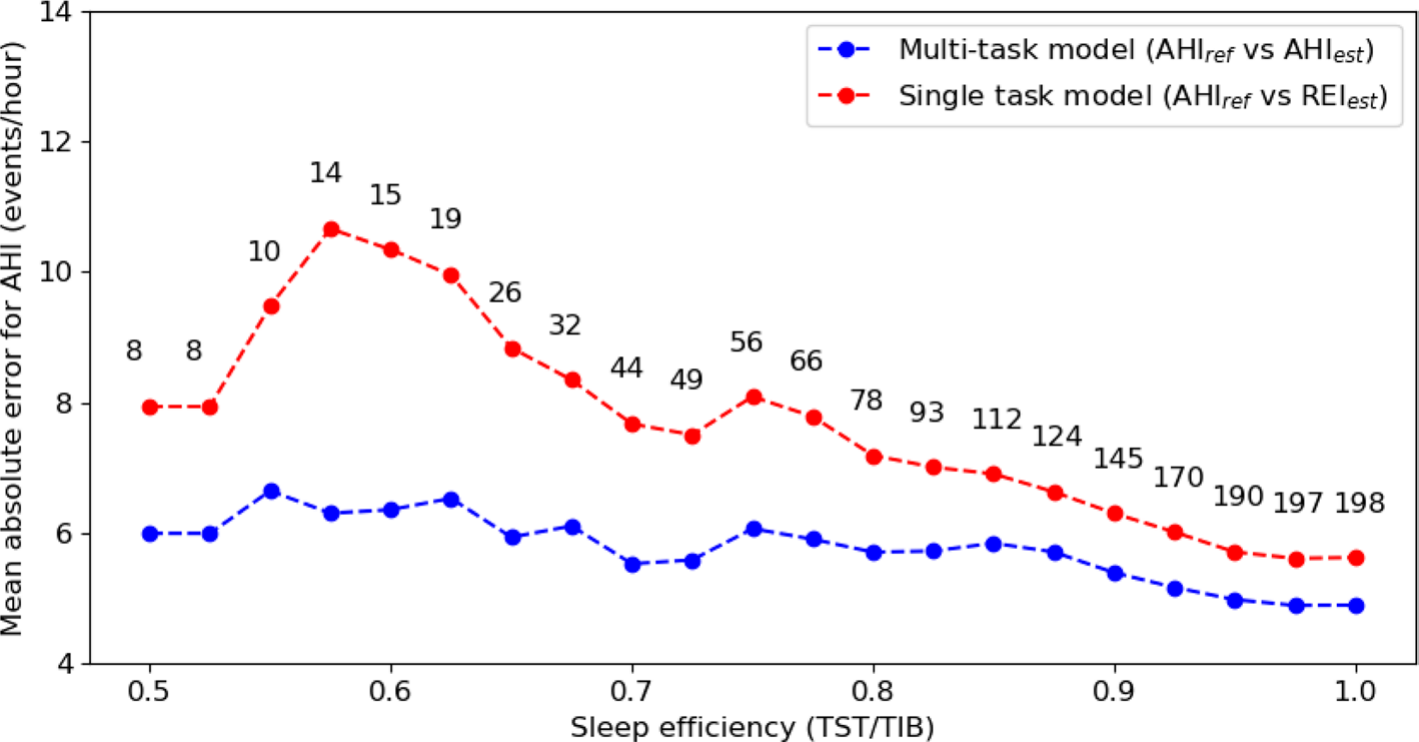
Mean absolute error between AHI_ref_ and AHI_est_ for the model with sleep–wake classification, and between AHI_ref_ and REI_est_ for the model without sleep–wake classification as a function of sleep efficiency. Each data point in the figure represents the MAE calculated from subjects with sleep efficiency lower than or equal to the corresponding value. Additionally, the number above each point indicates the total number of subjects included in the respective analysis. *TST:* total sleep time, *TIB:* total time in bed

Figures 6 and 7 present the Spearman’s correlation coefficients and MAE for AHI estimation obtained with the multi-task model, which includes sleep–wake classification (AHI_ref_ versus AHI_est_), and with the single-task model, which does not include sleep–wake classification (AHI_ref_ versus estimated respiratory event index or REI_est_), for a varying threshold of sleep efficiency (as scored from PSG). Performance with the multi-task model is always superior to that of the single-task model, in particular for patients with a sleep efficiency below 60% (*R* of 0.861 with multi-task model, *R* of 0.746 with single-task model). The performance converges as the sample increases to include subjects with higher sleep efficiency (more than half of the subjects had a sleep efficiency over 80%).

## Discussion

The aim of our study was to develop an algorithm capable of jointly detecting SDB events and classifying sleep–wake stages using RR intervals and RE as inputs, in a single integrated model. Using this multi-task approach, we sought to achieve accurate AHI estimation without relying on manual sleep–wake annotations or on a separate model for the sleep–wake classification task. Our algorithm exhibited a substantial correlation coefficient of 0.891 for AHI estimation across the entire dataset of 198 subjects with a wide range of SDB severities.

To benchmark our results with studies reported in literature, we restricted the comparison to studies using TST, either estimated directly from PSG, or which used a separate sleep–wake algorithm to obtain the TST which was then used to calculate AHI. For instance, Olsen et al. [14] reported an *R* of 0.910 for AHI estimation. Hafezi et al. [19] used deep learning models with features extracted from respiratory related movements recorded by a three-dimensional accelerometer, achieving an *R* of 0.84. The comparison between our work and these studies is limited by the fact that they used TST calculated from the sleep stages scored from PSG, and not from the inputs they used for SDB event detection. Errors in the estimation of TST, e.g., by using surrogate inputs such as in our study, will likely contribute to a lower AHI estimation performance. Unfortunately, to the best of our knowledge, there are very few studies that used the same surrogate inputs to both estimate TST and to detect SDB events. Notably, Papini et al. [20] employed a deep learning model on features from wrist‑worn reflective photoplethysmography and achieved an *R* of 0.67. In another study, Kwon et al. [21] applied CNN-LSTM network on images from impulse-radio ultra-wideband radar obtaining an *R* of 0.97. And it is also difficult to compare these studies because of the different datasets and input signals.

In our previous work, the single-task SDB event detection model could not rely on automatic sleep–wake classification, which meant that TST could not be estimated. In turn, that meant that the number of events per hour was calculated based on the time in bed (TIB), as the so-called respiratory event index (REI). We hypothesized that such an approach would underperform in comparison with AHI from PSG, in particular in patients with low sleep efficiency (lower amount of sleep relative to the TIB). Using TST estimated from the same input signals as for SDB event detection, led to an overall increase in AHI estimation performance when compared with AHI from PSG, particularly visible for patients with a sleep efficiency under 60%. This improvement has clinical significance, given that several studies have reported a high prevalence (39–55%) of insomnia symptoms among patients with SDB [16–18]. Expecting a substantial number of patients with (suspected) SDB to also present low sleep efficiency, our approach can yield more accurate AHI estimation results without the additional complexity of measuring TST by other means.

When comparing both approaches, we also found that the AHI and SDB severity of a few subjects with low sleep efficiency were overestimated by the model without sleep–wake classification, even when using the (comparably) longer TIB as the denominator for AHI computation. Subsequent investigation revealed that the single-task model also contained more false positive events than our multi-task model, primarily because wake periods were not excluded, resulting in the retention of false positive events during these periods. In contrast, our multi-task model, besides providing a more accurate estimation of the denominator of AHI, i.e., TST, also correctly removed false positive events during (detected) wake periods, further contributing to its improved performance. Nevertheless, it is relevant to acknowledge that such an approach may present issues in cases where sleep periods are erroneously detected as wake, leading to the removal of true positive events and possibly leading to an underestimation of AHI even if TST is overall reasonably well estimated. The comparison of both models also highlighted a limitation of our study, namely the somewhat insufficient representation of subjects with a low range of sleep efficiency. Although the MAE exhibited large differences between both methods for sleep efficiency below 60%, the somewhat similar correlation coefficients hint towards a lack of representative examples of subjects with simultaneously short TST (in relation to TIB) while presenting a wide range of SDB severity. Future studies should investigate this, possibly with a dedicated data collection effort, focusing on individuals presenting simultaneously a high pretest probability of SDB, and at the same time, symptoms of insomnia, or at least, short sleep time.

Another advantage of the present approach is that the model only requires RR intervals and respiratory effort as input. The present study is limited in that these were measured with arguably obtrusive sensors, such as ECG and thoracic RIP belts. However, noteworthy advancements in physiological sensing technology have suggested that both the RR intervals (or other measures of interbeat interval duration) and respiration (or surrogates thereof) can be acquired through more unobtrusive means. These encompass bed sensors [22, 23], chest-worn sensors [24, 25], and even wrist-worn photoplethysmography sensors [26, 27]. These sensors have demonstrated their capability to accurately capture the relevant physiological signals, suggesting that models such as the one of the present study could be feasibly applied in such settings for sleep–wake classification and apnea/hypopnea event classification. Future work should investigate whether the present model can be used with inputs acquired from such sensors.

The event detection rates might seem low in comparison with the relatively high accuracy in AHI estimation. This reason might be that cardiorespiratory patterns associated with SDB events do not necessarily coincide (in time) with the period of reduction of airflow; in fact, often they will only be visible after the actual SDB events, as sudden changes in respiratory patterns, or as the cardiac changes associated with the sympathetic discharges which often occur as the events resolve. It is clear that these post-event detections would count towards a correct estimation of AHI, but because they do not overlap with the periods coinciding with the cessation or reductions of airflow based on which the events are scored, they would not be counted as true positives, but instead as false positive, while the SDB events would be considered false negatives. This would simultaneously contribute to a reduction in the sensitivity and positive predictive value, but not to a reduction in AHI estimation performance.

For the model architecture, it seems logical that the SDB event detection part would work better if it were aware of the decisions of the sleep–wake classification, but the performance was worse when moving the shared part to the later layers, which might be attributed to the fact that both sleep and SDB event training targets are set to 1, potentially leading to an increased occurrence of false positives for SDB event detection. This should be further analyzed with a larger dataset.

## Conclusion

In conclusion, our study presented an algorithm for integrated SDB event detection and sleep–wake classification using RR intervals and RE. Besides offering the potential of surrogate SDB monitoring, the multi-task model allowed accurate AHI estimation without the need for manual sleep–wake annotation or additional sleep detection methods or other sensor modalities. In comparison with a similar method that did not use sleep–wake classification and relied instead on the TIB or TRT (TRT could potentially be even longer than TIB), the observed improvements in AHI estimation, particularly for subjects with low sleep efficiency, may have relevant clinical implications. Future research should focus on further validating this approach on cohorts with more subjects with low sleep efficiency, and finally validating the model with unobtrusive sensors more suitable for long-term monitoring at home.

## Methods

### Dataset

For this study, a total of 198 subjects, aged 18 and older, and not undergoing continuous airway pressure therapy, were obtained from the SOMNIA database [28]. No specific selection criteria were imposed based on body mass index (BMI), sex, or AHI. All subjects participated in routine PSG monitoring at the Kempenhaeghe Center for Sleep Medicine, located in Heeze, the Netherlands, between June 2017 and November 2017. A summary of their demographic and clinical characteristics is presented in Table 2.

**Table 2:** Subject characteristics

Male/female	121/77
Age, years	50.1 ± 14.8 (18–86)
BMI, kg/m^2^	27.2 ± 4.8 (18.6–45.2)
AHI, events/hour	18.0 ± 18.4 (0–108.4)
AHI < 5	48
5 < AHI < 15	67
15 < AHI < 30	46
AHI > 30	37

Numbers per subject are presented as mean ± SD over subjects

As part of the recommended set of sensors for in-lab PSG, the montage included ECG, recorded using modified lead II configuration with electrodes from Kendall (Ashbourne, Ireland), and RE, acquired using a Sleepsense (Elgin, USA) RIP belt mounted around the thorax.

Sleep stages and SDB events (obstructive, central and mixed apneas, and hypopneas) were scored based on the PSG by qualified sleep technicians, following the guidelines provided by the American Academy of Sleep Medicine (2015 rules). Specifically, hypopneas were scored using as confirmation rule, the presence of an SpO_2_ desaturation of at least 3% or the presence of a cortical arousal.

### Signal pre-processing and segmentation

The pre-processing and segmentation procedures closely followed the methodology of our previous work [15]. Specifically, for the ECG signals, R-peaks were initially detected using an algorithm based on nonlinear transformation and a simple peak-finding strategy [29]. Subsequently, a post-processing algorithm was employed to precisely localize the QRS complexes and eliminate artifacts [30]. Additionally, the method proposed by Mateo and Laguna [31] was applied to address ectopic beats: periods containing artifacts or ectopic beats were marked with a value of 0 for exclusion. The resulting RR intervals were then subjected to linear interpolation and resampled at a frequency of 4 Hz.

Similarly, RE signals were resampled at a rate of 4 Hz to ensure consistency with the RR time series. During the resampling process, high-frequency noise (> 2 Hz) was eliminated. Additionally, a Butterworth high-pass filter (order = 3) with a cut-off frequency of 0.05 Hz was utilized to remove low-frequency noise from the RE signals.

Next, all signals were segmented into 5-min segments with an overlap of 2 min. Subsequently, “soft” min–max normalization was applied to the RR interval and RE time series of each segment, with the minimum and maximum values set to the 5th and 95th percentiles, respectively. Finally, the RR and RE data of each segment were stacked to form an input bivariate vector of dimensions 1200 samples × 2 channels.

The scoring of SDB events was mapped into segments with the same 5-min length and overlap of 2 min. Each sample in the segment corresponds to a one-second period, set to 1 to indicate an SDB event of any type (obstructive, central, or mixed apneas, hypopneas) occurring during that sample, and to 0 during periods of normal breathing. This resulted in a vector of dimensions 300 samples × 1 label for each segment.

Similarly, annotations of sleep stages were mapped into segments with the same length and overlap to facilitate simultaneous learning of both tasks by the model, but at a sampling frequency of 1/30 Hz, corresponding to the epoch duration of the sleep stages scored from PSG. Each 30 s sample in the segment that corresponded to the sleep stage Wake was assigned a label of 0, while samples corresponding to any sleep stage (N1, N2, N3 or REM) were assigned a label of 1. Accordingly, each segment was represented with a sleep label vector of 10 labels (10 epochs × 1 label).

The two labels (SDB events, and wake/sleep stages) represent our training targets for the multi-task model. Notably, all segments occurring during the periods at the beginning and at the end of the recording when the lights were on (as annotated in the PSG) were removed.

### Multi-task deep learning model

**Fig. 8:**
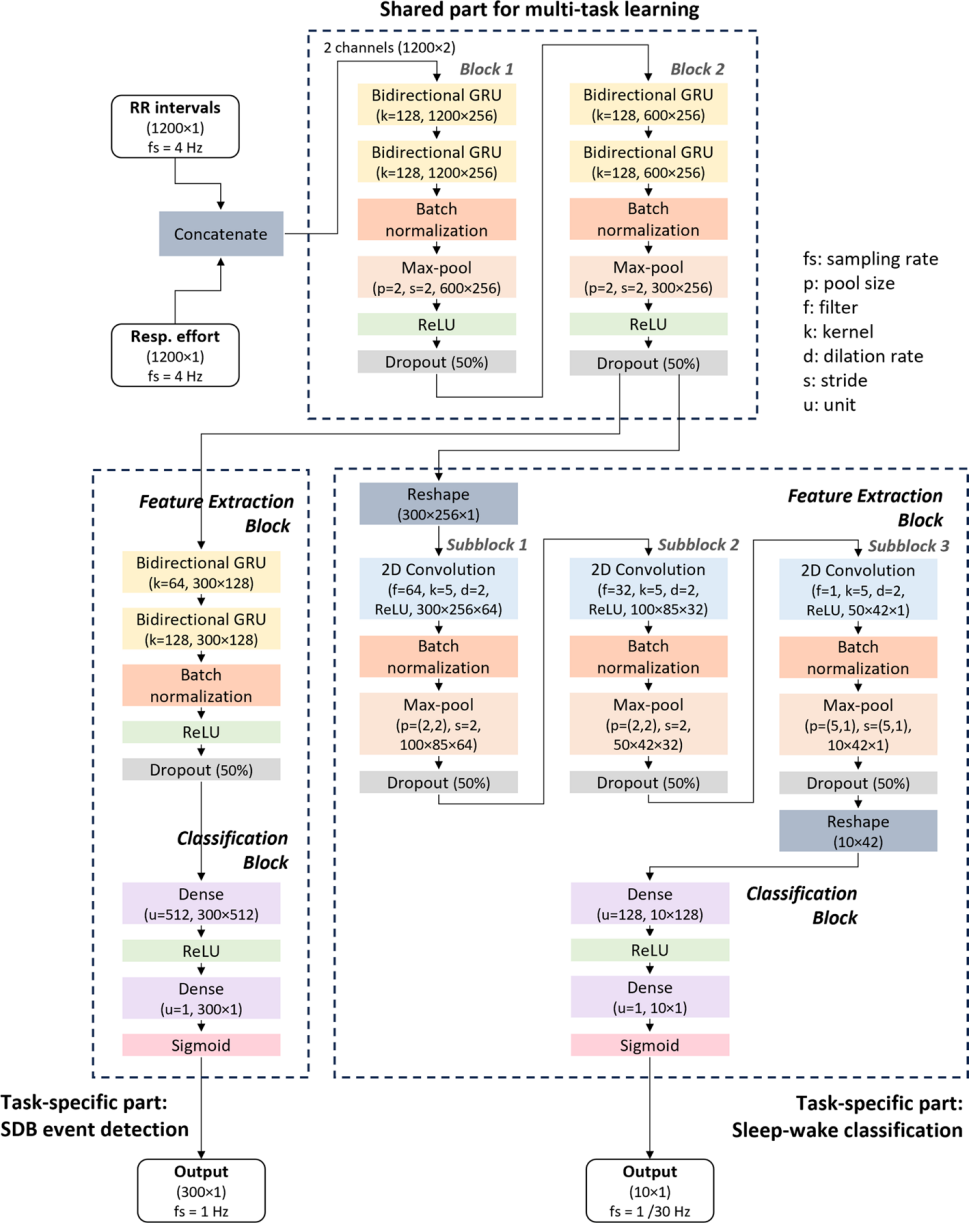
Multi-task model architecture; the parameters and output shape of each layer were presented together with the name of layer, the output shape of the layers which do not change the shape were not shown

The multi-task deep learning model architecture illustrated in Fig. 8 consists of three main components: a shared part for both tasks, a specific part for SDB event detection, and another specific part for sleep–wake classification. The architecture was determined experimentally. The shared part was designed to learn common latent representations relevant to both tasks. It comprised two blocks, which were analogous to the feature extraction blocks employed in prior studies [14, 15]. Each block consisted of two layers of bidirectional gated recurrent units (GRU), a batch normalization layer, a max-pool layer, an activation layer utilizing the rectified linear unit (ReLU) activation function, and a dropout layer.

The task-specific part for SDB event detection consisted of a feature extraction block and a classification block. The feature extraction block encompassed two layers of bidirectional GRUs, a batch normalization layer, an activation layer using the ReLU activation function, and a dropout layer. Subsequently, the classification block consisted of a dense-connected layer employing the ReLU activation function and another dense-connected layer utilizing the sigmoid activation function to generate the output.

Similarly, the task-specific part for sleep–wake classification included a feature extraction block combined with three subblocks. Each subblock was composed of a 2-dimensional convolution layer with ReLU activation, a batch normalization layer, a max-pool layer, and a dropout layer. Additionally, two reshape layers were incorporated at the beginning and end of the feature to adjust the shape for input and output. The classification block for sleep–wake classification mirrors that of event detection.

### Model training

Training was performed with an Adam optimizer with a learning rate of 0.001 and a weight decay of 0.0001, and a batch size of 128. The model initialization was conducted using Xavier uniform initializer. Given the imbalance between apnea/hypopnea events and normal periods, sample weighting was implemented, with a weight of 10 assigned to apnea/hypopnea events and a weight of 1 assigned to normal breathing periods. Binary cross-entropy was used as the loss function for both tasks and the final loss was set to the sum of the losses of the two tasks.

To mitigate the risk of overfitting, we used kernel regularization, dropout and incorporated an early stopping mechanism: training was terminated when no decrease in the validation loss was observed in 10 epochs.

In addition to the multi-task model, we also trained a separate single-task model, as utilized in our previous study [15], to allow a direct comparison with our proposed multi-task model for AHI estimation.

### Training, validation, and testing data splitting

**Fig. 9:**
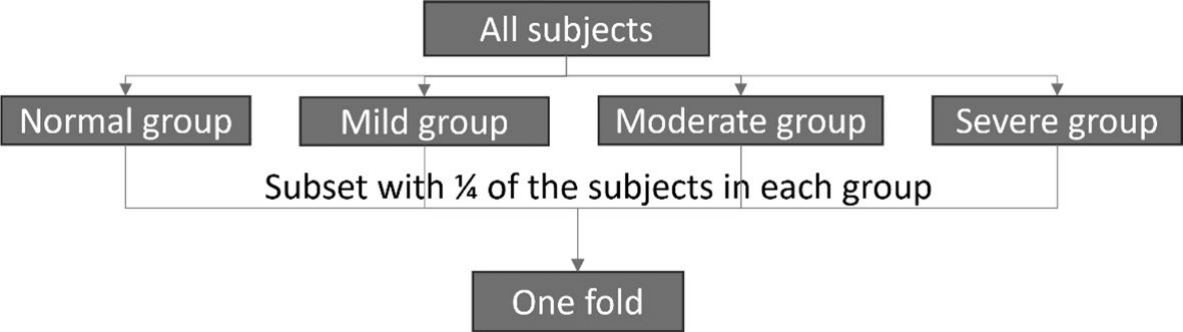
Process to form one fold. All subjects were first divided into four severity groups, each group was then randomly divided into four subsets with ¼ of the subjects in the group; One subset from each group was used to form one fold

The study implemented a fourfold cross-validation approach to effectively leverage the available dataset for model evaluation and training. The division of the dataset into four folds was done through a stratified random split method, designed to maintain a balanced distribution of subjects across all levels of SDB severity. Initially, all subjects were categorized into four distinct SDB severity groups based on their AHI values from the annotation: normal (AHI < 5), mild SDB (5 < AHI < 15), moderate SDB (15 < AHI < 30), and severe SDB (AHI > 30) [1]. Subsequently, subjects within each severity group were randomly assigned to four subsets, and one subset was chosen from each group to form one fold of the cross-validation procedure. The cross-validation procedure is depicted in Fig. 9. During each iteration, one fold was designated as the testing set, while the remaining three folds were merged and further partitioned into training and validation sets.

**Fig. 10:**
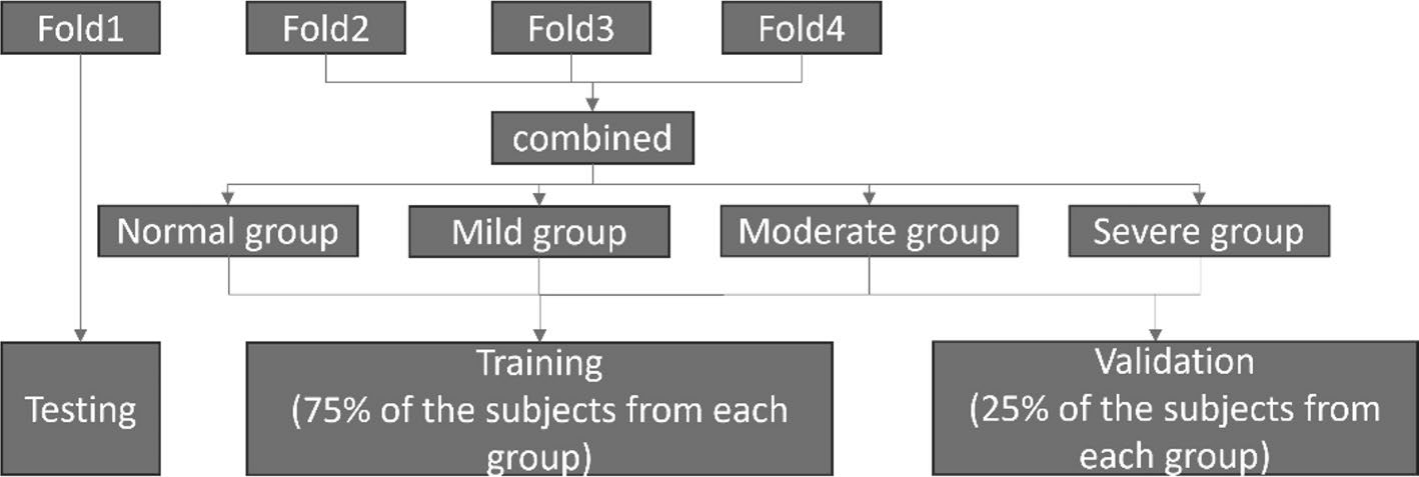
Training, validation, and testing set for the first iteration. One fold was used for testing, while the remaining three folds were combined again and then divided into four SDB severity groups, and 75% of subjects from each group were used for training while the remaining 25% were used for validation

To create the training and validation sets, the three folds were combined and subsequently divided into the four SDB severity groups. 75% of the subjects from each group were taken for training, and the remaining 25% for validation. To ensure that samples from the same subject are only assigned to one set (either the training, validation, or testing set), all samples from each subject adhere to their corresponding subject’s assignment. Figure 10 illustrates the configuration of the training, validation, and testing sets in the first iteration of the four fold cross-validation process. This process was repeated for each cross-validation iteration, with a different fold designated as the testing set in each round, while the training and validation sets were assembled as described.

In each iteration of the cross-validation process, the model was fitted using the training set, while the validation set was utilized for early stopping and to determine the optimal decision threshold. The results obtained on each recording of the testing set for each cross-validation iteration were finally combined to assess the overall performance for all subjects in the dataset.

### Performance evaluation

Performance was evaluated for different tasks, namely sleep–wake classification, SDB event detection, AHI estimation, and SDB severity classification.

Sleep–wake classification was evaluated in terms of epoch-per-epoch agreement between the predictions of the model, and the sleep stages manually scored from PSG. The model outputs a value between 0 and 1; to obtain a binary classification, we automatically selected, on the validation set of each cross-validation iteration, the threshold that yielded the best F1 score for sleep (as positive class, label 1) versus wake classification. This threshold was then used, on the same iteration, to obtain the binary classification on the segments of the recordings of the testing set. Performance was finally evaluated by comparing the classification for the six 30-s epochs of the 3 min on the middle of each 5-min segment, discarding the outer 2 min which overlapped neighboring segments. Agreement was assessed by means of the Cohen’s kappa coefficient of agreement, F1 scores, sensitivity, and specificity between the predictions and the reference class from PSG. Additionally, we calculated the Spearman’s correlation coefficient and MAE between the estimated TST and the PSG reference TST for each recording.

Similarly, for SDB event detection, we evaluated the middle 3 min of each segment. The segments were transformed into events based on a methodology described in previous studies [14, 15]. Specifically, we assigned a one-second sample to an event if the model output for SDB event detection surpassed a threshold. Samples scored as part of an SDB event during a period detected as Wake by the assessment above were assigned to ‘normal breathing’ periods. The threshold used to decide whether a sample was part of an SDB event was automatically determined by maximizing the F1 score for event detection on the validation set of each cross-validation iteration. Consecutive samples classified as SDB events were combined to form a single event. True positives, false positives and false negatives were then computed according to the same rules as the previous study [15]. Finally, sensitivity, precision, and F1 score were used as performance measures for SDB event detection.

AHI_est_ was estimated by dividing the number of detected SDB events by the estimated TST, while the reference AHI_ref_ was obtained by dividing the number of PSG scored SDB events by the reference TST, also from PSG. Bland–Altman analysis, scatter plots, and Spearman’s correlation coefficient (*R*) were employed to assess the agreement between AHI_ref_ and AHI_est_.

To evaluate the performance of SDB severity classification, we categorized subjects into different classes based on their AHI_ref_ and AHI_est_ values, namely as normal (AHI < 5), mild SDB (5 < AHI < 15), moderate SDB (15 < AHI < 30), and severe SDB (AHI > 30). To address potential biases near the class boundaries, the NBL technique was employed [32, 33]. This technique involved assigning subjects with AHIs near the class boundaries to two severity classes, and the estimated class was considered correctly classified if it fell into either one of the two classes. The near-boundary zones from Pee et al. [33] were used in this study. The confusion matrix, accuracy, and Cohen’s Kappa were computed both with and without the NBL technique.

Finally, to assess the added value of sleep–wake classification with our multi-task model, we compared the AHI estimation performance with that obtained with a single-task model where the model outputs only SDB event detection, but no estimation of the periods of sleep and wake. In the latter case, the ratio of events per hour is estimated not based on TST (which is not available as an output of the model), but rather, on the TIB. This index, often referred to as REI, is commonly used in applications or systems where a measurement of sleep (and TST) is not available, and we expect AHI and REI to diverge especially in recordings where the subject spends a relatively large amount of the time in bed awake. Accordingly, we calculated the Spearman’s correlation coefficient and MAE between AHI_ref_ and AHI_est_ (with the multi-task model) and between AHI_ref_ and REI_est_ (with the single-task model) across a varying range of sleep efficiencies. Specifically, we applied a varying threshold on sleep efficiency, obtained from PSG as the ratio between TST and TIB, and calculated the performance for all subjects with a sleep efficiency lower than that threshold.

## Data Availability

The SOMNIA data used in this study are available from the Sleep Medicine Centre Kempenhaeghe upon reasonable request. The data can be requested by presenting a scientific research question and by fulfilling all the regulations concerning the sharing of the human data. The details of the agreement will depend on the purpose of the data request and the entity that is requesting the data (e.g., research institute or corporate). Each request will be evaluated by the Kempenhaeghe Research Board and, depending on the request, approval from independent medical ethical committee might be required. Access to data from outside the European Union will further depend on the expected duration of the activity; due to the work required from a regulatory point of view, the data is less suitable for activities that are time critical, or require access in short notice. Specific restrictions apply to the availability of the data collected with sensors not comprised in the standard PSG set-up, since these sensors are used under license and are not publicly available. These data may however be available from the authors with permission of the licensors. For inquiries regarding availability, please contact Merel van Gilst (M.M.v.Gilst@tue.nl).

## Abbreviations

SDB: Sleep disorder breathing
AHI: Apnea–hypopnea index
PSG: Polysomnography
ECG: Electrocardiogram
EDR: ECG-derived respiration
SpO_2_: Oxygen saturation
RE: Respiratory effort
RIP: Respiratory inductive plethysmography
TRT: Total recording time
TIB: Time in bed
TST: Total sleep time
REI: Respiratory event index
NBL: Near boundary double-labeling
MAE: Mean absolute error
BMI: Body mass index
SD: Standard deviation
GRU: Gated recurrent units
ReLU: Rectified linear unit
